# On the identity of *Eucephalobus oxyuroides* (de Man, 1876) Steiner, 1936 (Rhabditida, Cephalobidae), with an updated taxonomy of the genus and notes about its phylogeny

**DOI:** 10.21307/jofnem-2020-061

**Published:** 2020-07-28

**Authors:** Joaquín Abolafia, Reyes Peña-Santiago

**Affiliations:** Departamento de Biología Animal, Biología Vegetal y Ecología, Universidad de Jaén, Campus “Las Lagunillas”, s/n. 23071 Jaén, Spain

**Keywords:** 18S rDNA, 28S rDNA, Description, Key, Molecular analysis, Morphology, Phylogeny, SEM, Taxonomy

## Abstract

The identity of *Eucephalobus oxyuroides* is discussed after studying two Iberian populations that fit the original description of the species. A new characterization is consequently provided as follows: 0.49 to 0.70 mm long body, lip region continuous with the adjoining body and bearing short probolae prongs, neck 132 to 158 µm long, spermatheca 23 to 47 µm long or 1.0 to 1.8 times the corresponding body diameter, post-vulval uterine sac 40 to 54 µm long or 2.0 to 2.5 times as long as the body diameter, *V*  =  58 to 64, female tail conical elongate with very acute terminus (60-79 µm, *c* = 7.1-10.0, *c*′ = 4.9-5.8), male tail conical (36-49 µm, *c* =10.9-14.3, *c*′ = 2.4-3.4) with an elongate acute mucro, and spicules 21 to 22 µm long. Previous records of the species are revised. An emended diagnosis of the genus is proposed, and its taxonomy is updated with a list of species, key to their identification and illustrations. The evolutionary relationships of *Eucephalobus*, as derived from the analyses of 18S and 28S rDNA fragments, reveals that it occupies a basal position within the subfamily Cephalobidae.

*Cephalobus oxyuroides* was described by de Man (1876) from the Netherlands, and named according to its apparent resemblance with *Cephalobus oxyuris* (Bütschli, 1873). The latter was regarded by Thorne (1937) as a junior synonym of *Panagrolaimus rigidus* (Schneider, 1866) Thorne, 1937. Its original description was based on five specimens, three females and two males, which were characterized by their acute lips, conoid-elongate female tail, and male bearing a long mucro at tail end and spicules with rounded and manubrium bent ventrad (Fig. 1A, H). Unfortunately, other relevant traits such as length and morphology of the post-vulval uterine sac were not mentioned. de Man (1881, 1984) studied additional material of *C. oxyuroides* and provided new illustrations of the female (Fig. 1B-G), which show a long, poorly defined post-vulval structure that might have been a post-vulval uterine sac, but that probably was interpreted erroneously. Later, several authors (Örley, 1880; Cobb, 1893a, 1893b; Micoletzky, 1914, 1917, 1921; Rahm, 1928, 1929) recorded the species and/or provided some data about it, but in general only fragmentary information was given (see further analysis below).

**Figure 1:**
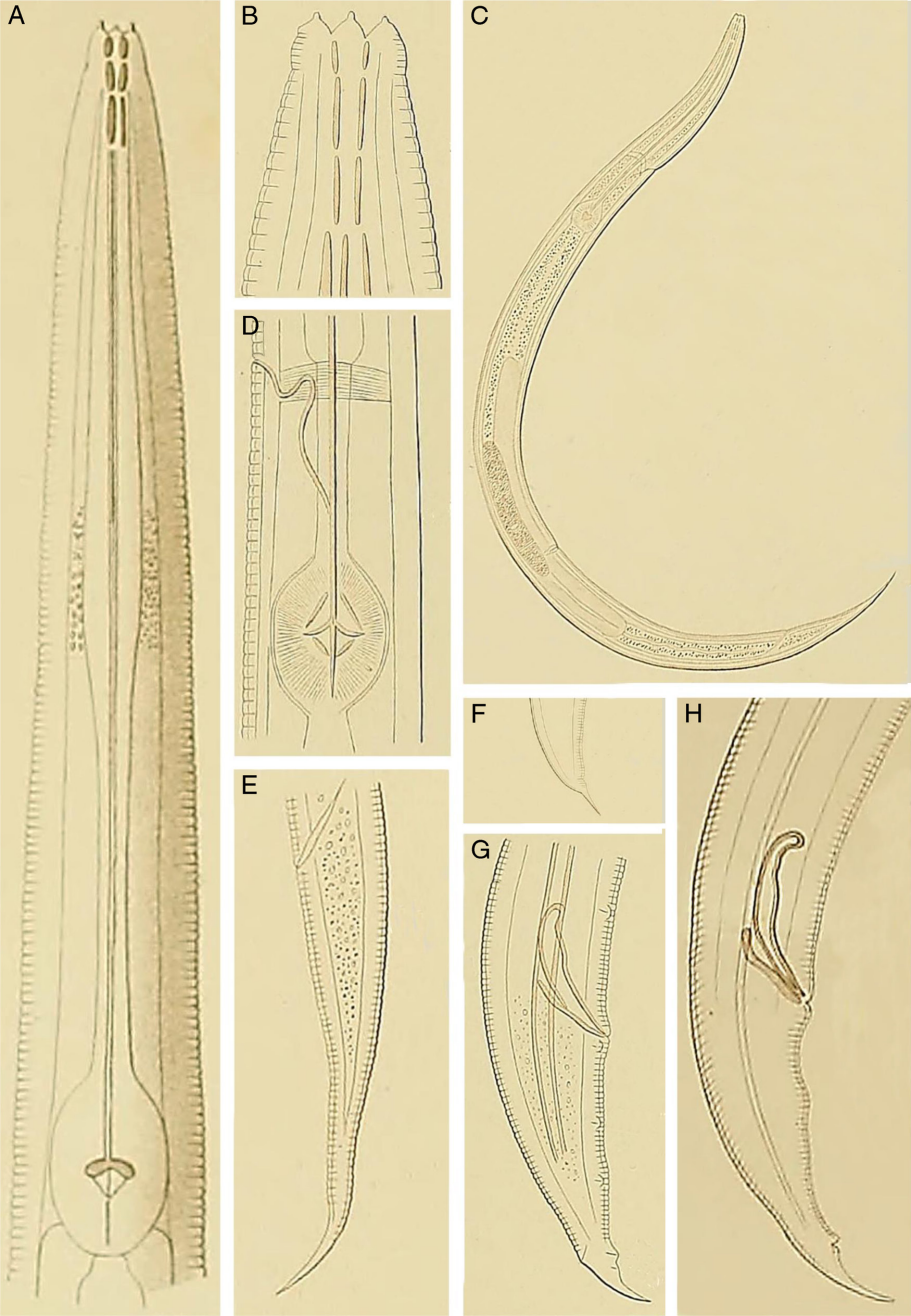
Facsimile reproduction of the original drawings of *Cephalobus oxyuroides* (A, H from de Man (1881) and B-G from de Man (1984)). (A) Neck; (B) Stoma; (C) Entire female; (D) Excretory pore; (E) Female posterior end; (F-H) Male posterior end.

Steiner (1936a, 1936b) proposed the new genus *Eucephalobus* Steiner, 1936a, with *E. oxyuroides* as its type species. Since then, this taxon has been repeatedly mentioned in the literature, especially in contributions by Thorne (1937), Schuurmans-Stekhoven and Teunissen (1938), Allgén (1950), Meyl (1953a, 1953b, 1955), Heyns (1961), Andrássy (1967), Ivanova (1968), Mavljanov (1976), Nesterov (1979), Boström (1984, 1993) Abolafia and Peña-Santiago (2002), Iliev et al. (2003), and Kim et al. (2017). Nevertheless, the species was never subject of a detailed morphological study for comparative purposes, and the true identity of some records therefore remains questionable.

Two *E. oxyuroides* populations from the Iberian Peninsula were collected in the course of a nematological survey conducted in natural areas. Their study revealed that they are very similar and tentatively regarded here as conspecific with the original material described by de Man. This contribution pursues their characterization, discusses the identity of previously reported populations, and updates the taxonomy of the genus.

## Materials and methods

### Nematode extraction and processing

Nematodes were obtained from soil samples using a modified Baermann (1917) funnel technique, killed by heating, fixed in a 4% formalin solution, transferred to pure glycerine following the technique of Siddiqi (1964), and mounted on glass slides for observation.

### Light microscopy

Observations were made using a Leitz Laborlux S (Leitz, Wetzlar, Germany) microscope and a Nikon Eclipse 80i (Nikon, Tokio, Japan) with differential interference contrast optics. Measurements were taken and drawings were made with the Leitz microscope using a drawing tube (*camera lucida*). Pictures were taken with the Nikon microscope and a Nikon Digital Sight DS-U1 camera. Micrographs of the same structure were combined using Adobe^®^ Photoshop^®^ CS. The terminology used for the morphology of stoma and spicules follows the proposals by De Ley et al. (1995) and Abolafia and Peña-Santiago (2017), respectively.

### Scanning electron microscopy (SEM)

Specimens preserved in glycerine were selected for observation under SEM according to the methods of Abolafia (2015). They were hydrated in distilled water, dehydrated in a graded ethanol-acetone series, critical point dried, coated with gold, and observed with a Zeiss Merlin microscope (5 kV) (Zeiss, Oberkochen, Germany).

### Phylogenetic analyses

For phylogenetic relationships, analyses were based on 18S and 28S rDNA gene sequences available in GenBank. The sequences were aligned using the ClustalW alignment tool implemented in the MEGA7 (Kumar et al., 2016). The ambiguously aligned parts and divergent regions were found using the online version of Gblocks 0.91b (Castresana, 2000) and were removed from the alignments using MEGA7. The best-fit model of nucleotide substitution used for the phylogenetic analysis was statistically selected using jModelTest 2.1.10 (Darriba et al., 2012). Phylogenetic trees were generated with the Bayesian inference method using MrBayes 3.2.6 (Ronquist et al., 2012). *Drilocephalobus* sp. (AY284679) and *Teratolobus* sp. (KJ652552) were chosen as outgroups for the 18S and 28S rDNA trees, respectively. Analysis under the GTR + I + G model was initiated with a random starting tree and run with the Markov chain Monte Carlo for 1 × 10^6^ generations. The tree was visualized and saved with FigTree 1.4.4 (Rambaut, 2018).

## Results

**Figure 2:**
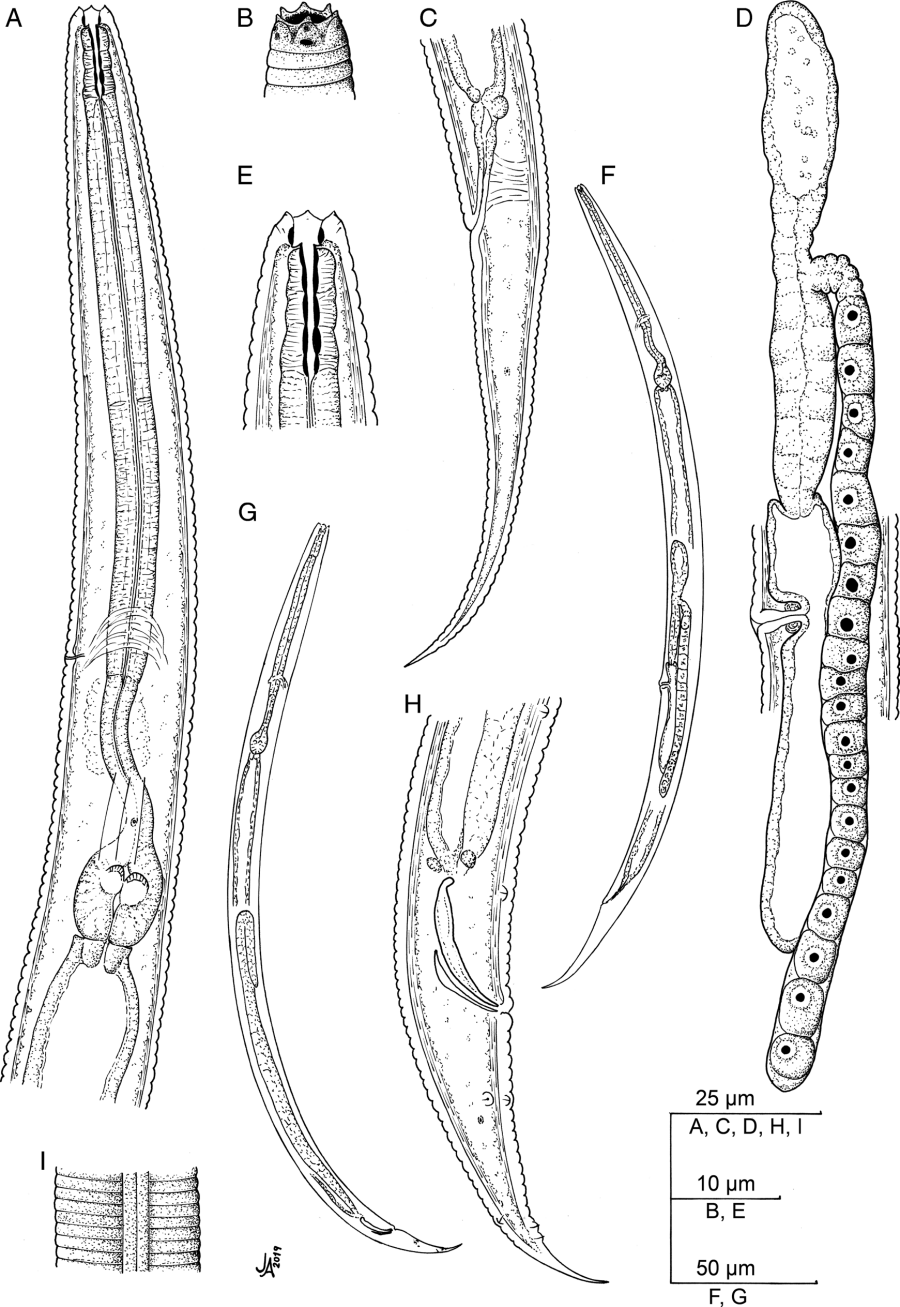
*Eucephalobus oxyuroides* (de Man, 1876) Steiner, 1936a (line drawing). (A) Neck; (B) Lip region; (C) Female posterior end; (D) Female reproductive system; (E) Stoma; (F) Entire female; (G) Entire male; (H) Male posterior end; (I) Lateral field.

**Figure 3:**
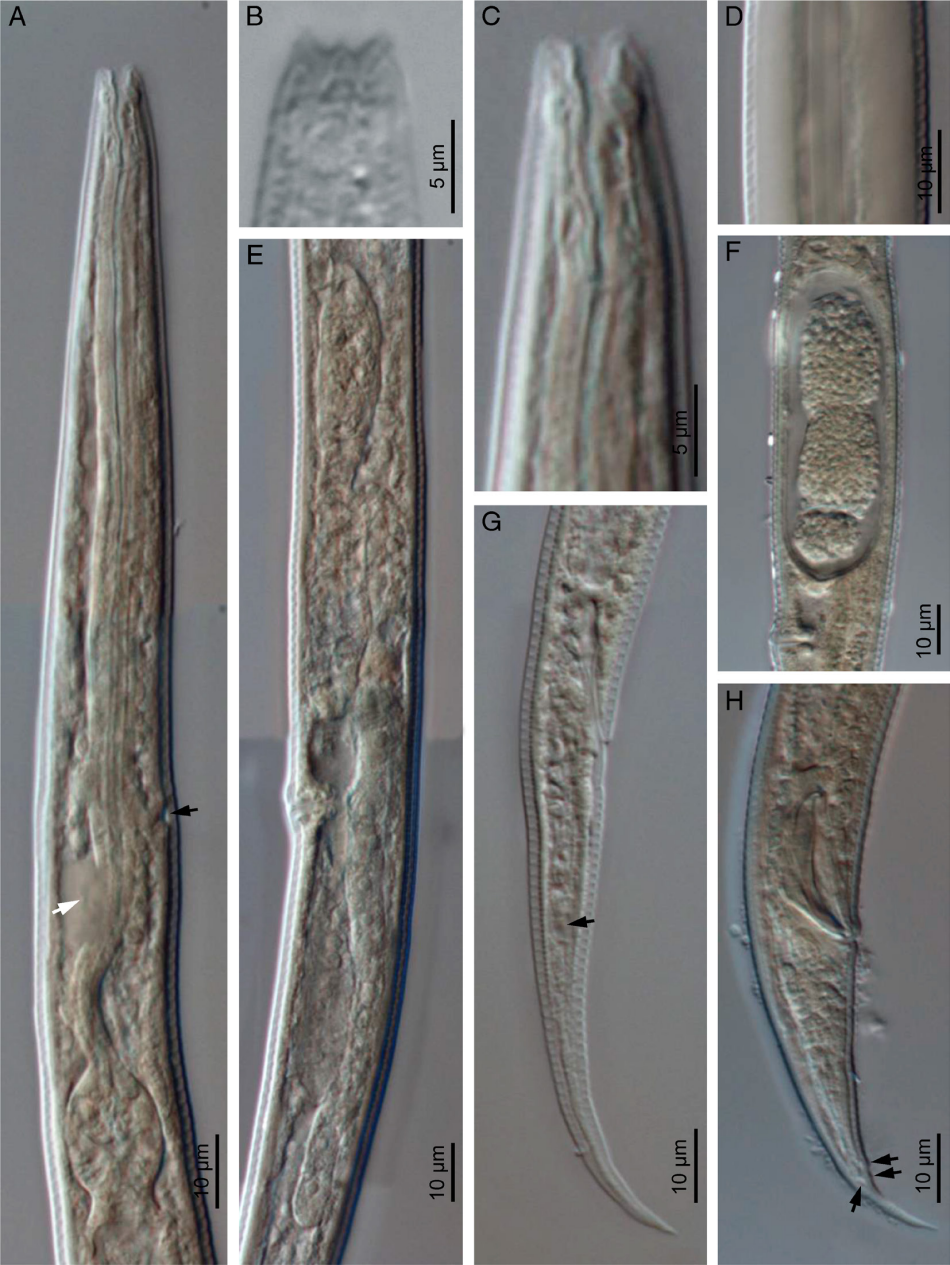
*Eucephalobus oxyuroides* (de Man, 1876) Steiner, 1936a (light microscopy). (A) Neck (black arrow pointing to the excretory pore, white arrow pointing to the deirid); (B) Lip region; (C) Stoma; (D) Lateral field; (E) Female reproductive system; (F) Uterine egg; (G) Female posterior end (arrow pointing the phasmid); (H) Male posterior end (arrows pointing genital papillae).

**Figure 4:**
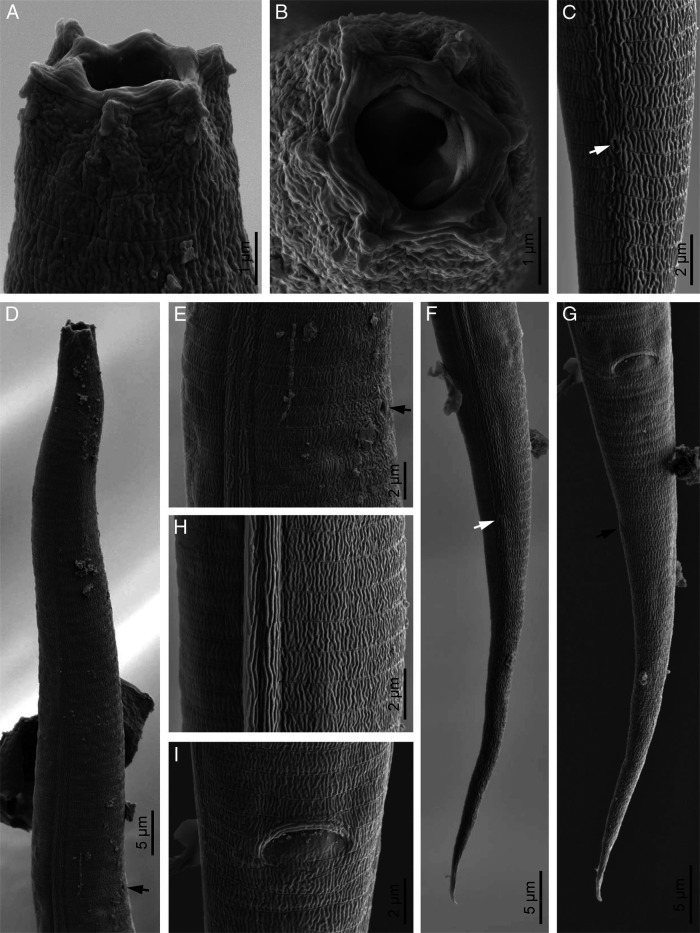
*Eucephalobus oxyuroides* (de Man, 1876) Steiner, 1936a (scanning electron microscopy). (A, B) Lip region in lateral and frontal view, respectively; (C) Female phasmid (arrow); (D) Neck (arrow pointing the excretory pore); (E) Excretory pore (arrow); (F, G) Female posterior end in lateral and ventral view, respectively (arrow pointing the phasmid); (H) Lateral field; (I) Anus.

*Eucephalobus oxyuroides* (de Man, 1876) Steiner, 1936a (Figs. 2-4).

Material examined from Navarra (Spain): 11 females and 9 males, in good condition.

### Measurements

Measurements are provided in Table 1.

**Table 1.** Morphometrics of *Eucephalobus oxyuroides* (de Man, 1876) Steiner, 1936a from Spain.

Province	Navarra		Jaén
Habitat	Oak forest		Whine forest
Sex	Female	Male	Female
*n*	11	9	2
Body length	610 ± 44.7 (554-695)	567 ± 35.7 (513-629)	493, 602
*a*	28.7 ± 1.9 (26.6-32.4)	29.1 ± 2.9 (25.1-32.9)	21.4, 26.2
*b*	4.2 ± 0.3 (3.9-4.7)	4.0 ± 0.2 (3.8-4.3)	3.5, 4.3
*c*	8.8 ± 0.6 (8.0-10.0)	12.7 ± 1.0 (10.9-14.3)	7.1, 8.4
*c'*	5.3 ± 0.3 (5.0-5.8)	2.9 ± 0.3 (2.4-3.4)	4.9, 5.5
*V*	60.3 ± 1.1 (58-64)	–	58, 60
Lip region width	6.0 ± 0.0 (6)	6.0 ± 0.0 (6)	6, 7
Stoma length	13.5 ± 0.5 (13-14)	13.2 ± 0.8 (12-14)	14, 14
Pharyngeal corpus length	86.2 ± 3.4 (82–91)	84.9 ± 5.6 (77-97)	88, 86
Isthmus length	28.2 ± 3.0 (23-31)	27.2 ± 2.9 (22-32)	21, 25
Bulbus length	17.0 ± 1.2 (14-18)	16.4 ± 0.9 (15-18)	17, 15
Pharynx length	131 ± 5.4 (123–140)	128.6 ± 8.0 (119-145)	126, 126
Nerve ring – anterior end	95.1 ± 4.0 (89–103)	93.0 ± 5.3 (84-101)	100, 102
Excretory pore – anterior end	98.3 ± 43.8 (94-107)	95.4 ± 5.2 (86-102)	103, 101
Deirid – anterior end	114 ± 3.5 (106-117)	110.4 ± 6.5 (97-119)	119, 117
Neck length (stoma + pharynx)	145 ± 5.4 (137-153)	141.8 ± 8.3 (132-158)	140, 140
Body diameter at midbody	21.4 ± 2.1 (19-25)	19.7 ± 2.2 (17-23)	23, 23
Lateral field width	3.5 ± 0.6 (3-4)	3.4 ± 0.5 (3-4)	4, 4
Anterior ovary/testis length	135 ± 17.4 (116-160)	115.6 ± 14.9 (99-144)	118, 110
Spermatheca length	33.4 ± 7.9 (23-47)	–	44, 55
Post-vulval uterine sac length	47.1 ± 4.0 (40-54)	–	48, 57
Vulva – anterior end	368 ± 25.3 (328-405)	–	285, 363
Rectum length	18.9 ± 1.4 (16-20)	5.0 ± 0.7 (4-6)	20, 20
Anal body diameter	13.1 ± 0.8 (12-14)	15.7 ± 1.1 (14-18)	14, 13
Tail length	69.5 ± 5.1 (60-79)	44.9 ± 4.2 (36-49)	69, 72
Spicules length	–	21.4 ± 0.5 (21-22)	–
Gubernaculum length	–	13.7 ± 1.9 (12-16)	–

**Notes:** Measurements in μm and in the form: mean ± standard deviation (range) where appropriate. Demanian indices (de Man, 1881): *a*, body length/body diameter; *b*, body length/pharynx length; *c*, body length/tail length; *c′*, tail length/anal body diameter; *V* = (distance from anterior region to vulva/body length) ×100.

### Description

#### Adult

They are moderately slender to slender (*a* = 25-33) nematodes of small size, 0.51 to 0.70 mm long. Upon fixation, habitus is nearly straight or somewhat curved ventrad. Cuticle is 2 µm thick, bearing transverse striation, and annuli is 2 µm wide. Lateral field is 3 to 4 µm broad, occupying 13 to 22% of mid-body diameter. Lip region is continuous with the adjacent body. Lips are conoid with protruding, conical labial and cephalic sensilla; primary and secondary axils are similar to each other. Labial probolae are three, biacute, bearing short acute prongs, each one fused to its more proximal lip. A fine incisure occurs between each labial probola and the adjacent lip pair. Amphids are very small, oval, located at the base of lateral lips. Stoma is cephaloboid, 2.2 to 2.3 times the lip region diameter long: cheilostom with strongly refringent, bar-shaped rhabdia, posteriorly thicker; gymnostom very reduced, with small rhabdia; stegostom consists of poorly refringent rhabdia, and metastegostom bearing dorsal acute rhabdion (dorsal tooth). Pharynx is also cephaloboid: pharyngeal corpus is subcylindrical, 2.6 to 3.8 times the isthmus length, with procorpus and metacorpus not clearly separate; isthmus is comparatively thin, slightly tapering to its junction with basal bulb; the latter is ovoid with both valvular apparatus and posterior haustrulum well developed. Cardia is small, surrounded by intestinal tissue. Nerve ring is at 84 to 103 µm or 61 to 69% of neck length from the anterior end, surrounding the posterior part of metacorpus. Excretory pore is at 86 to 107 µm or 64 to 72% of neck length, at the level of metacorpus-isthmus junction, adjacent to the hemizonid. Deirids are posterior to excretory pore, at 97 to 119 µm or 73 to 83% of neck length, at the level of isthmus. Intestine is without distinct specializations, but with slightly thinner walls at cardiac part.

#### Female

Reproductive system is monodelphic-prodelphic. Ovary is 116 to 160 µm long, usually with a flexure at its distal portion. Oviduct is very short, slightly areolate; spermatheca is 23 to 47 µm long, 1.0 to 1.8 times the corresponding body diameter long. Uterus is 48 to 80 µm long, 2.5 to 3.5 times as long as body diameter, tubular, and sometimes containing uterine eggs (53-62 × 21-23 µm). Post-vulval uterine sac is swollen, 40 to 54 µm long, and 2.0 to 2.5 times as long as the body diameter. Vagina is 7 to 9 µm long, extending inwards over 35 to 42% of the body diameter. Vulva is slightly protruding. Rectum is short, 1.1 to 1.7 times the anal body width; three large gland-like cells are distinguishable around the intestine-rectum junction. Tail is conical-elongate with acute terminus. Phasmids are located at 19 to 23 µm or 28 to 33% of tail length from anus, and lateral fields terminating at phasmids.

#### Male

Reproductive system is monorchic, with testis reflexed ventrad anteriorly. Spicules are paired and symmetrical: manubrium is rounded, well developed and ventrad bent, short conoid calamus, and slightly curved ventrad lamina with acute tip in lateral view. Gubernaculum is well developed, slightly curved, about one-half of the spicule length, with thin corpus. Three small gland-like cells are distinguishable around the beginning of the cloaca. Tail is conical, slightly curved ventrad, ending in an elongate acute mucro. Genital papillae has seven pairs, two pre-cloacal and five post-cloacal: two pairs subventral at the middle of tail length, two ventral subterminal, and one lateral subterminal. One mid-ventral adcloacal papilla is present. Phasmids are at 21 to 24 µm or 46 to 55% of tail length from the cloacal aperture, close to middle genital papillae.

#### Other material examined

Two females examined from Acebeas (province of Jaén), with similar morphology and morphometry (Table 1) to the Navarra population, but one female is slightly smaller (493 µm long).

### Distribution

The Navarra population (northern Iberian Peninsula) was collected from soil of an oak (*Quercus robur* L.), near Pamplona. The two specimens were from Jaén (southern Iberian Peninsula) from soil of an oak (*Quercus faginea* Lam.) forest in Acebeas, Natural Park of Cazorla, Segura and Las Villas, province of Jaén.

## Discussion

### Proposal of a new concept of *E. oxyuroides*

Iberian specimens match well with the type material described by de Man (1876), but males show slightly larger general size (513-629 vs 447-514 µm long body) and slightly more anterior excretory pore (at 64-72 vs 59% of neck length from the anterior end). Unfortunately, details of post-vulval uterine sac, an important diagnostic character of the species, were not originally provided. Nevertheless, a few years later, de Man (1881) described additional material, including both females and males, and illustrated the female genital system with a long (about 2.5 times the body diameter) postvulval sac. These new specimens studied by de Man do not differ from the Iberian ones in any relevant morphological or morphometric trait, including the long post-vulval sac (2.5 vs 2.0-2.5 times the body diameter). Loof (1961) re-examined one female and one male of de Man’s material, but he was unable to provide any relevant morphological or morphometrical information about it, due to its bad condition according to this author. Thus, with due caution, it is herein assumed that Iberian and Dutch exemplars are conspecific.

Based on the material now examined, *E. oxyuroides* is characterized by its 0.49 to 0.70 mm long body, cuticle with 2 µm broad annuli, lip region continuous with the adjoining body and 6 µm wide, bearing short probolae, neck 132 to 158 µm long, spermatheca 23 to 55 µm long or 1.0 to 1.8 times the corresponding body diameter, uterus 48 to 80 µm long or 2.5 to 3.5 times as long as body diameter, post-vulval uterine sac 40 to 57 µm long or 2.0 to 2.5 times as long as the body diameter, vagina 7 to 9 µm long, *V* = 58 to 64, female tail conical elongate with very acute terminus (60-79 µm, *c* = 7.1-10.0, *c*′ =  4.9-5.8), male tail conical (36-49 µm, *c* = 10.9-14.3, *c*′ = 2.4-3.4) with an elongate acute mucro, and spicules 21 to 22 µm long.

### On the identity of previously known populations

The above description of *E. oxyuroides* allows analysis and discussion of the identity of previously recorded populations (Table 2).

**Table 2.** Compendium of *Eucephalobus oxyuroides* (de Man, 1876) Steiner, 1936a populations.

Reference	Country	*n*	Body length	*a*	*b*	*c*	*c*^*'*^	*V*	Stoma	Pharynx length	Neck length	Nerve ring − ant. end (%)	Excretory pore − ant. end (%)	Body width at midbody	Sper-matheca length	Post-vulval uterine sac / body diameter	Vulva-ant. end	Rectum length (female)	Anal body width	Tail length	Tail mucro	Spicules length	Gubernaculum length
1	Spain	13 females	493-695	21.4-32.4	3.5-4.7	7.1-10.0	4.9-5.8	58-64	13-14	123-140	137-153	61-69	65-72	19-25	23-47	2.0-2.5	285-405	16-20	12-14	60-79	Conoid elongate	–	–
		9 males	513-629	21.1-32.9	3.8-4.3	10.9-14.3	2.4-3.4	–	12-14	119-145	132-158	64-68	64-71	17-23	–	–	–	–	14-18	36-49	Conoid elongate	21-22	12-16
2	The Netherlands	5 males	447-514**	20.0-21.8**	3.1-3.2**	10.4-11.9**	2.5-2.7**	–	12**	125-149**	145-161**	?	59**	22-24**	–	–	–	–	16-17**	33-45**	Conoid elongate	22**	12*
3	The Netherlands	? females	700	20.0-25.0	4.0	8.0-9.0	5.1*	61*	?	?	?	66*	65*	?	?	2.5*	?	?	?	?	Conoid elongate	–	–
		? males	640	20.0-25.0	4.0	12.0-13.0	2.4*	–	?	?	?	?	?	?	–	–	–	–	?	?	Conoid elongate	?	?
4	Hungary	1 female	620	23.8**	3.9**	8.9**	?	56**	?	?	160	?	?	26	?	?	350**	?	?	70	?	?	?
		2 males	450-500	20.8-21.4**	3.6-3.8**	11.1-11.3**	?	–	?	?	125-132	?	?	21-24	–	–	–	–	?	40-45	Conoid elongate	–	–
5	Australia	1 female	640	17.8**	4.5*	7.1**	5.3**	51	10**	?	143**	?	69**	36**	?	?	?	?	17**	90	?	–	–
6	Switzerland	1 male	342	19.0	2.8	6.0	?	–	11	112**	122**	?	?	16**	–	–	–	–	?	57**	?	?	?
7	Austria	1 female	459	22.0	3.1	8.1	?	61	8**	141**	149**	57**	?	21**	?	?	?	?	10**	57**	?	–	–
8	Rumania	5 females	500-565	19.4-21.8	3.7-4.1	6.8-7.8	?	56-59	?	?	?	?	?	?	?	?	?	?	?	?	?	–	–
		1 male	527	19.0	3.0	17.8	?	–	?	?	176**	?	?	28**	?	?	?	?	?	30**	?	?	?
9	Austria	19 females	450-610	18.6-29.0	3.0-4.9	6.8-18.0	5.0*	58-72	?	?	?	?	?	?	?	?	?	?	?	?	Short conoid	–	–
		11 males	410-520	17.7-24.0	3.5-5.9	9.6-12.8	?	–	?	?	?	?	?	?	–	–	–	–	?	?	?	?	?
10	Brazil	1 female	1926	21.4	4.2-5.0	12.4	?	60	?	?	?	?	?	?	?	?	?	?	?	?	?	–	–
		1 male	1485	29.0	4.9	16.5	?	–	?	?	?	?	?	?	–	–	–	–	?	?	?	45	?
11	UK	1 female	650	19.0	3.9	8.3	5.2*	60	?	?	167**	?	?	34**	?	?	390**	?	?	78**	Conoid elongate	–	–
		1 male	550	21.0	3.5	11.6	2.7*	-	?	?	?	?	?	29**	–	–	–	–	?	47**	Conoid elongate	?	?
12	D. R. Congo	1 female	356	22.2	3.4	8.0	?	?	?	?	104**	?	?	16**	?	?	?	?	?	45**	?	–	–
13	Sweden	1 female	787	28.4	4.8	16.0	?	65	?	?	164**	?	?	28**	?	?	512**	?	?	49**	?	–	–
14	Norway	1 female	800	38.1	3.8	12.3	?	?	?	?	213	?	?	21	?	?	?	?	?	65	?	–	–
		1 male	787	35.7	4.2	15.7	?	?	?	?	213	?	?	22	?	?	?	?	?	50	?	?	?
15	Italy	? females	467-683	16.8-22.4	3.3-4.3	7.7-9.0	?	59-65	?	?	?	?	?	?	?	?	?	?	?	?	?	–	–
16	South Africa	? females	410-460	20.0-22.0	3.8-3.9	8.7-13.1	?	63	?	?	?	?	?	?	?	?	?	?	?	?	?	–	–
17	Mongolia	1 female	540	20.0	3.4	10.7	3.6	60	?	?	158**	?	?	27**	?	1.4	326**	?	?	50**	Short conoid	?	?
		1 male	490	20.0	2.6	13.5	2.2	–	?	?	188**	?	?	25**	–	–	–	–	?	36**	Short conoid	–	–
18	Hungary	? females	450-500	20.0-23.0	4.0-4.2	8.5-9.5	5.0-6.0	60-62	12	?	?	?	74-77	?	?	0.8*	?	?	?	?	Conoid elongate	–	–
19	Tadjikistan	? females	460	20.0-22.0	3.4-3.8	8.0	4.3*	60	?	?	?	?	?	?	?	?	276**	?	?	58**	?	–	–
20	Uzbekistan	1 female	450	22.2	39.4^1^	9.1	?	62	?	?	?	?	?	20**	?	?	279**	?	?	49**	?	–	–
21	Russia, Turkmenistan, Moldavia	? females	750-900	22.0-24.0	3.8-4.4	7.0-9.6	?	58-62	?	?	?	?	?	?	?	?	?	?	?	?	?	–	–
		? males	700-800	24.0-25.2	5.0-5.6	7.0-10.7	?	–	?	?	?	?	?	?	–	–	–	?	?	?	?	?	?
22	Russia	1 female	353	17.6	4.0	5.7	?	50	?	?	88**	?	?	20**	?	?	?	?	?	62**	?	–	–
		2 males	480-488	16.0-20.0	3.0-3.5	10.0	2.9*	-	13	131*	144	65*	66*	30-	–	–	–	–	18*	48-49**	Conoid elongate	20	12
23	Spain	42 females	450-700	17.0-23.0	3.0-5.0	6.5-9.0	6.0*	56-72	?	?	?	?	?	?	?	?	?	?	?	?	Conoid elongate	–	–
		3 males	450-650	19.0-28.0	3.0-5.0	7.0-11.0	2.3*	–	?	?	?	?	?	?	–	–	–	–	?	?	Absent	?	?
24	Sweden	? females	?	?	?	?	?	?	?	?	?	?	?	?	?	1.0-3.0	?	?	?	59-73	Conoid elongate	–	–
25	Brazil	74 females	370-650	18.0-32.0	3.3-5.0	5.0-8.5	7.3*	55-63	10-14	140*	108-170	61*	65*	19-23*	16-32*	1.1-1.3*	?	20-24	13*	57-105	Short conoid	–	–
		28 males	390-600	21.0-35.0	3.2-4.2	14.6-19.8	2.5*	–	10-14	?	117-144	?	?	21*	–	–	–	–	14*	24-37	Seta-like or wart-like	14-20	9-12
26	Austria	6 females	510-560	19.0-22.0	3.4-3.7	7.1-8.0	4.0-5.0	60–62													?	–	–
		1 male	520	20.0	3.4	9.5	?	–								1.0-1.5					?	23	12
27	Turkey	1 female	609	23.0	4.0	8.7	4.4	63	12	154	166**	61	63	27	34	1.2	?	19	16**	70	Short conoid	–	–
		1 male	525	28.0	3.9	16.4	1.9	–	11	133	144**	63	65	19	–	–	–	–	?	32	Short conoid	17	10
28	Spain	4 females	524-535	21.4-27.6	3.5-3.7	8.4-8.9	4.2-5.3	60-64	9-14	143-150	156-161**	54-64	54-67	19-25	26-36	0.7-1.7	317-338	18-20	12-15	60-64	Short conoid	–	–
29	Bulgaria	5 females	588-729	19.0-26.0	3.8-4.3	5.9-7.4	5.0-6.8	57-61	?	127-136	?	?	66-74	17-22	?	0.5	?	13-20	?	32-36	?	–	–
		3 males	436-556	25.0-30.0	3.2-3.8	10.9-15.7	1.9-2.3	–	?	124-139	?	?	64-69	16-17	?	?	?	–	?	30-36	?	14-21	8-10
30	Hungary	? females	380-740	18.0-28.0	3.3-5.0	6.0-10.0	4.2-5.8	56-66	10-14	120-150	?	?	74-78	?	?	< 1.0	?	?	?	70-90	?	–	–
		? males	300-620	20.0-28.0	3.2-4.2	14.0-18.0	?	–	10-14	120-150	?	?	74-78		–	–	–	–	?	?	?	14-20	9-11

**Notes:** References: 1. Present paper, 2. de Man (1875), 3. de Man (1881, 1984), 4. Örley (1880), 5. Cobb (1893a), 6. Stefan´ski (1914), 7. Micoletzky (1914), 8. Micoletzky (1917), 9. Micoletzky (1921), 10. Rahm (1928, 1929), 11 Thorne (1937), 12. Schuurmans-Stekhoven and Teunissen (1938), 13. Allgén (1950), 14. Allgén (1953), 15. Meyl (1953a, 1953b), 16. Heyns (1961), 17. Andrássy (1964), 18. Andrássy (1967), 19. Ivanova (1968), 20. Mavljanov (1976), 21. Nesterov (1979), 22. Mukhina (1981), 23. Monreal and Campoy (1982), 24. Boström (1984), 25. Rashid et al. (1984), 26. Gerber (1991), 27. Boström (1993), 28. Abolafia and Peña-Santiago (2002), 29. Iliev et al. (2003), 30. Andrássy (2005), 31. Kim et al. (2017). *Measurements from drawings; **Measurements from other measurements; ?Unknown measurement; –Character absent; ^1^Probably a mistake.

### Material fitting type material

Those populations or specimens examined by Örley (1880) from Hungary, Thorne (1937) from UK, and Boström (1984) from Sweden match well with the type and others described by de Man (1876, 1881, 1984), having in common a lip region with short labial probolae, excretory pore and nerve ring at metacorpus-isthmus junction, long post-vulval uterine sac *ca.* twice as long as the body diameter while lacking constrictions and septae (only provided in Boström, 1984), and male tail with elongate acute ventral curved mucro.

Mukhina (1981) described a population of *Heterocephalobus nannus* (Steiner, 1936a) Andrássy, 1967 from Primorsky Krai (Russia). Although only the male was illustrated, both the morphological and the morphometrics of this population fit well the diagnosis of *E. oxyuroides*. This it is herein regarded as belonging to this species.

### Material not fitting type material

Several records of the species significantly differ from the aforementioned in one or several relevant features. Thus:

1. Cobb (1893a, 1893b) described but did not illustrate *Cephalobus similis* from Australia, later regarded as identical with *E. oxyuroides* by Andrássy (1967). The female genital system of this species was considered as ‘probably double and symmetrical,’ a feature totally incompatible with the cephalobid pattern.
2. Rahm (1928, 1929) described but did not illustrate *Cephalobus oxyuroides* var. *brasiliensis* from Brazil on the basis of two excessively large specimens (body 1.92 mm in female and 1.49 mm in male). This material certainly does not belong to *Eucephalobus*.
3. Several features of the Mongolian population studied by Andrássy (1964), who provided Demanian indices and illustrations of the tail in both sexes, suggest that it is not conspecific with the type due to a shorter post-vulval uterine sac (1.4 times the body diameter) and female tail (*c*′ = 3.6), male tail with very short mucro, and spicules lacking manubrium bent ventrad. It might not be a member of *Eucephalobus*.
4. The Hungarian females studied by Andrássy (1967) possess high labial probola prongs, excretory pore located at anterior part of isthmus, and post-vulval sac shorter than body diameter. They resemble *E. compsus* (Steiner, 1935) Thorne, 1937.
5. Monreal and Campoy (1982) recorded *E. oxyuroides* from northern Spain, provided Demanian indices and illustrated the tail of both sexes. The male tail lacks a distinct mucro, thus this material does not belong to this species. The lip region was not described, but these Iberian specimens might be members of the genus *Pseudacrobeles* Steiner, 1938.
6. The Brazilian population studied by Rashid et al. (1984) is distinguishable by its short post-vulval sac (about one body diameter), male with seta-like or wart-like mucro, and spicules lacking a ventrad bent manubrium. As suggested by De Ley et al. (1993), it resembles *Pseudacrobeles variabilis*.
7. Gerber (1991) described (but did not illustrate) six females and one male from Austria characterized by having post-vulval sac 1.0 to 1.5 times the body diameter. This feature is not compatible with *E. oxyuroides*.
8. Boström (1993) studied one female and one male from Turkey characterized by having excretory pore apparently located at metacorpus-isthmus junction, post-vulval sac 1.2 times the body diameter and divided in two sections, and male tail bearing a short mucro. Although the author did not provide illustrations, available information suggests that this material resembles *E. hooperi* Marinari-Palmisano, 1967 or *E. compsus*.
9. The four Iberian females recorded by Abolafia and Peña-Santiago (2002) have their excretory pore and nerve ring situated at level of metacorpus, post-vulval sac more or less swollen and slightly divided in two sections, with septae. These specimens better fit *E. compsus*.
10. The very short post-vulval sac (one-half of body diameters) of the Bulgarian specimens examined by Iliev et al. (2003) is not compatible with *E. oxyuroides.*
11. Kim et al. (2017) described *E. oxyuroides* from South Korea, but this population resembles *E. hooperi* much more, due to the morphology of post-vulval sac (divided into two regions, proximally tubular and distally swollen) and tail in both sexes.

#### Material with unverifiable identity

Several records of the species lack key information that might allow confirmation of their precise identity. This is the case of the populations/specimens described by Micoletzky (1914, 1917, 1921; Austria, Romania), Stefański (1914; Switzerland) Schuurmans-Stekhoven and Teunissen (1938; former Zaire), Allgén (1950, 1953; Sweden, Norway, respectively), Meyl (1953a, 1953b; Italy), Heyns (1961; South Africa), Ivanova (1968; Tajikistan), Mavljanov (1976; Uzbekistan), and Nesterov (1979; Moldova).

#### Other records

The simple mention of *Eucephalobus oxyuroides* has been reported in many contributions that did not provide any other data, namely Schneider (1923; Germany), De Coninck (1930; Belgium), Franz (1943; Austria), Andrássy (1952, 1960, 1973; Hungary, China, respectively), Meyl (1954, 1955; Germany), Gadea (1967, 1984; Spain), Loof (1971; Norway), Palomo (1977; Spain), Coomans (1989; Belgium), Bussau (1990; Germany), Armendáriz et al., (1996; Spain), Al Banna and Gardner (1996; USA), Bushmakiu et al. (2000; Moldova), Ferenc (2001; Hungary), Lišková and Renčo (2007; Slovakia), Carrión and Desgarennes (2012; Mexico), Gagarin (2018; Vietnam), and Shurkurovich and Akvarovna (2018; Uzbekistan).

### Phylogeny and systematics of *Eucephalobus*

#### Diagnosis (emended)

Cephalobidae, Cephalobinae: they are small nematodes, 0.36 to 1.00 mm long, cuticle bearing transverse striation, lateral field with three longitudinal incisures, lip region continuous with the adjacent body and consisting of conoid lips, primary and secondary axils showing similar morphology, biacute labial probolae bearing prongs of variable length, each one fused at its more proximal lip, oval or rounded amphids located at the base of lateral lips, stoma and pharynx are cephaloboid, nerve ring is surrounding the metacorpus or the isthmus, excretory pore is at level of metacorpus or isthmus, female reproductive system is monodelphic-prodelphic, cephaloboid, ovary is usually with a flexure at its distal portion, oviduct is very short, spermatheca is well developed, post-vulval uterine sac is 0.5 to 2.5 times the body diameter, rectum is slightly longer than the anal body diameter, female tail is conical-elongate, conoid, or subcylindrical with seta-like, conoid or ragged mucro, phasmids are at anterior part of tail, male reproductive system is monorchic with testis reflexed anteriorly, spicules are slightly ventral curved, gubernaculum is almost straight, male tail is conical with seta-like, conoid or ragged mucro, genital papillae has seven pairs, two pre-cloacal and five post-cloacal, and phasmids are at mid-length of tail.

### List of species

**Figure 5:**
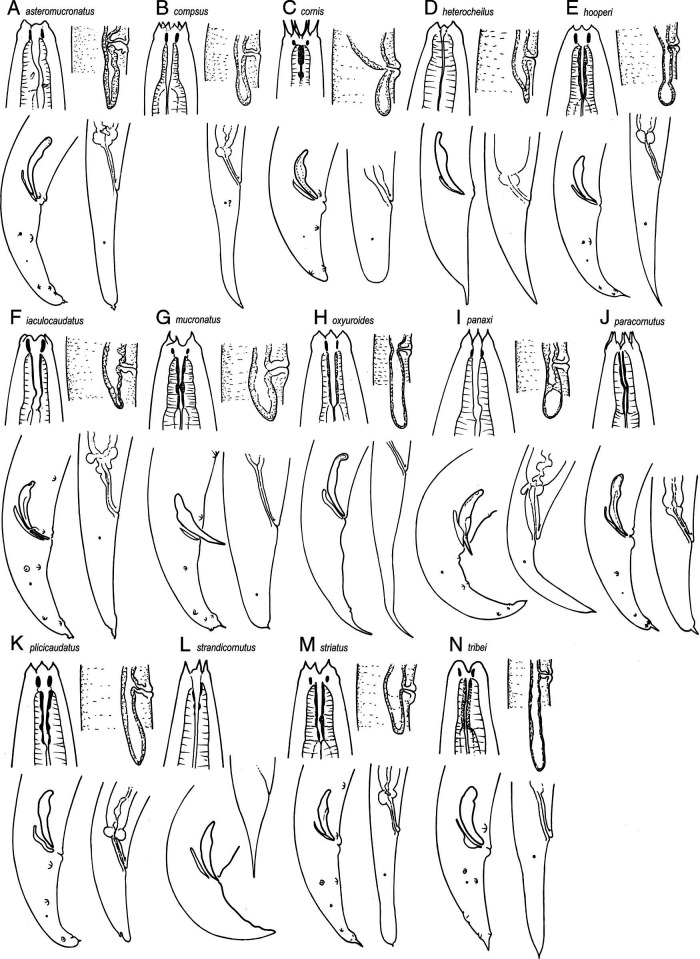
Lip region, post-vulval uterine sac, male and female tails of the species of the genus *Eucephalobus* Steiner, 1936a including *species inquirendae*. Based on original descriptions except E (male tail) cf. Amirzadi et al. (2013); G (lip region), M (lip region, tails) cf. Andrássy (1967); H (post-vulval sac), M (post-vulval sac) cf. Boström (1984); H (female tail) cf. de Man (1984). Not to scale.

The genus *Eucephalobus* includes 13 valid species plus 2 *species inquirendae vel incertae sedis* (Fig. 5).

Type species:

*E. oxyuroides* (de Man, 1876) Steiner, 1936a.

= *Cephalobus oxyuroides* de Man, 1876.

= *Cephalobus (Eucephalobus) oxyuroides* de Man, 1876 (Schneider, 1939).

= *Cephalobus oxyuroides* f. *acaudata* Micoletzky, 1921.

= *Cephalobus similis* Cobb, 1893a.

= *Heterocephalobus nannus apud* Mukhina, 1981 *nec* Steiner (1936a), n. syn.

Other species:

*E. asteromucronatus* Bostrom and Holovachov, 2011.

*E. compsus* (Steiner, 1935) Thorne, 1937.

= *Neocephalobus compsus* Steiner, 1935.

*E. cornis* (Thorne, 1925) Andrássy, 1967.

= *Acrobeles (Acrobeloides) cornis* Thorne, 1925.

=  *Cephalobus cornis* (Thorne, 1925) Thorne, 1937.

*E. hooperi* Marinari-Palmisano, 1967.

= *Eucephalobus oxyuroides apud* Kim et al. (2017), *nec* de Man (1876), n. syn.

*E. iaculocaudatus* Bostrom and Holovachov, 2011.

*E. mucronatus* (Kozłowska and Roguska-Wasilewska, 1963) Andrássy, 1967.

= *Eucephalobus mucronatus* Kozłowska and Roguska-Wasilewska, 1963.

= *Cephalobus dubius* var. *apicata* Maupas, 1900.

= *Cephalobus persegnis* var. *apicata* Maupas, 1900 (Micoletzky, 1921).

= *Cephalobus striatus* f. *tubifera* sf. *typica* Micoletzky, 1921.

= *Cephalobus striatus* f. *microtubifera* Micoletzky, 1921.

= *Cephalobus persegnis apud* Rühm, 1956 *nec* Bastian (1865).

= *Eucephalobus arcticus* Loof, 1971.

*E. panaxi* Mukhina, 1990.

*E. paracornutus* de Coninck, 1943.

*E. plicicaudatus* Mukhina, 1970.

*E. strandicornutus* (Allgén, 1934) Andrássy, 1967.

= *Cephalobus strandicornutus* Allgén, 1934.

= *Cephalobus (Heterocephalobus) strandicornutus* Allgén, 1934 (Brzeski, 1960).

= *Heterocephalobus strandicornutus* (Allgén, 1934) Brzeski, 1961.

*E. striatus* (Bastian, 1865) Thorne, 1937.

= *Cephalobus striatus* Bastian, 1865.

= *Cephalobus (Eucephalobus) striatus* Bastian, 1865 (Schneider, 1939).

= *Cephalobus striatus* f. *atubifera* Micoletzky, 1921.

= *Cephalobus bursifer* de Man, 1876.

*E. tribei* Swart and Heyns, 1997.

*Species inquirendae vel incertae sedis:*

*E. heterocheilus* (Steiner, 1935) Andrássy, 1984 (see Comments).

= *Panagrolaimus heterocheilus* Steiner, 1935.

= *Heterocephalobus heterocheilus* (Steiner, 1935) Andrássy, 1967.

*E. setensis* Kannan, 1960 (see Comments).

Species transferred to other genus:

*E. nannus* Steiner, 1936a to *Panagrolaimus* (see Comments).

= *Tricephalobus nannus* (Steiner, 1936a) Steiner, 1936b.

= *Heterocephalobus nannus* (Steiner, 1936a) Andrássy, 1967.

### Comments on some species

The identity of *E. heterocheilus* raises some doubt. It is characterized by having acute lips and cephaloboid stoma as well as pharynx, but the female reproductive system lacks a spermatheca and its ovary is long, reaching the posterior part of the intestine, features better agreeing with *Panagrolaimus* Fuchs, 1930. The male posterior end, with a fine mucro curved dorsad, also resembles that present in some *Panagrolaimus* species. It is possible that this species was originally described from material belonging to more than one species. It is hence regarded here as *species inquirenda*.

Andrássy (1984) considered *E. setensis* to be a *species inquirenda*. Its original description was based on only one Indian female, characterized by a combination of features (lips not acute, wide stomatal lumen, indistinct genital system with very short uterus and without spermatheca or post-vulval sac, and elongate tail covered by short setae-like filaments, probably artifact) that does not fit the *Eucephalobus* pattern. Thus, it is herein regarded as *incertae sedis*.

Some relevant morphological traits of *E. nannus* suggest that it does not belong to this genus. Its long gymnostom and very short stegostom, pharynx with short slightly robust metacorpus and elongate isthmus, and conoid female tail better fit the pattern of the genus *Panagrolaimus* resembling for instance *P. verrucosus* Fuchs, 1930. Thus, it is transferred to this genus as *Panagrolaimus nannus* (Steiner, 1936a) n. comb.

### Key to species identification

(1a) Female tail conoid elongate with acute tip 2.

(1b) Female tail short conoid, subcylindrical or clavate with rounded tip, with or without mucro 7.

(2a) Female tail conoid tapering sharply from its middle *strandicornutus*.

(2b) Female tail conoid tapering uniformly until the end 3.

(3a) Post-vulval uterine sac *ca.* twice longer than body diameter 4.

(3b) Post-vulval uterine sac as long as or shorter than body diameter 5.

(4a) Probolae short but visibly acute; male tail usually with mucro as long as gubernaculum (rarely shorter); spicules elongate with small rounded manubrium and long calamus *oxyuroides*.

(4b) Probolae very reduced, obscure; male tail with very short mucro; spicules robust with wide rounded manubrium and short calamus *tribei*.

(5a) Lip region as wide as adjacent part of body; post-vulval uterine sac slightly constricted at its middle length *compsus*.

(5b) Lip region narrower that the adjacent part of body; post-vulval uterine sac wider at its middle length 6.

(6a) Post-vulval uterine sac with tubular proximal part and swollen distal part; male tail with elongate fine mucro; spicules with calamus as long as wide *hooperi.*

(6b) Post-vulval uterine sac not well differentiated in tubular and globular part, this latter with solid lumen; male tail with short mucro, thicker at it base; spicules with calamus longer than wide *panaxi*.

(7a) Female tail with warty or irregular mucro 8.

(7b) Female tail with smooth seta-like mucro or lacking mucro 9.

(8a) Nerve ring and excretory pore more anterior, at metacorpus level; female tail subcylindrical with mucro placed centrally on tip *asteromucronatus*.

(8b) Nerve ring and excretory pore more posterior, at isthmus level; female tail conical with mucro placed ventrally on tip *iaculocaudatus*.

(9a) Female tail conoid with rounded terminus or slightly clavate 10.

(9b) Female tail subcylindrical 11.

(10a) Female tail slightly clavate, with or without mucro *striatus*.

(10b) Female tail conoid lacking mucro *plicicaudatus*.

(11a) Female and male tails lacking mucro *cornis*.

(11b) Female and male tails usually with mucro 12.

(12a) Tail with long and robust mucro placed ventrally on tip; spicules 23 to 25 µm long *paracornutus*.

(12b) Tail with short and thin mucro placed centrally on tip, sometimes absent; spicules 25 to 33 µm long *mucronatus*.

### Notes about the phylogeny of *Eucephalobus*

**Figure 6:**
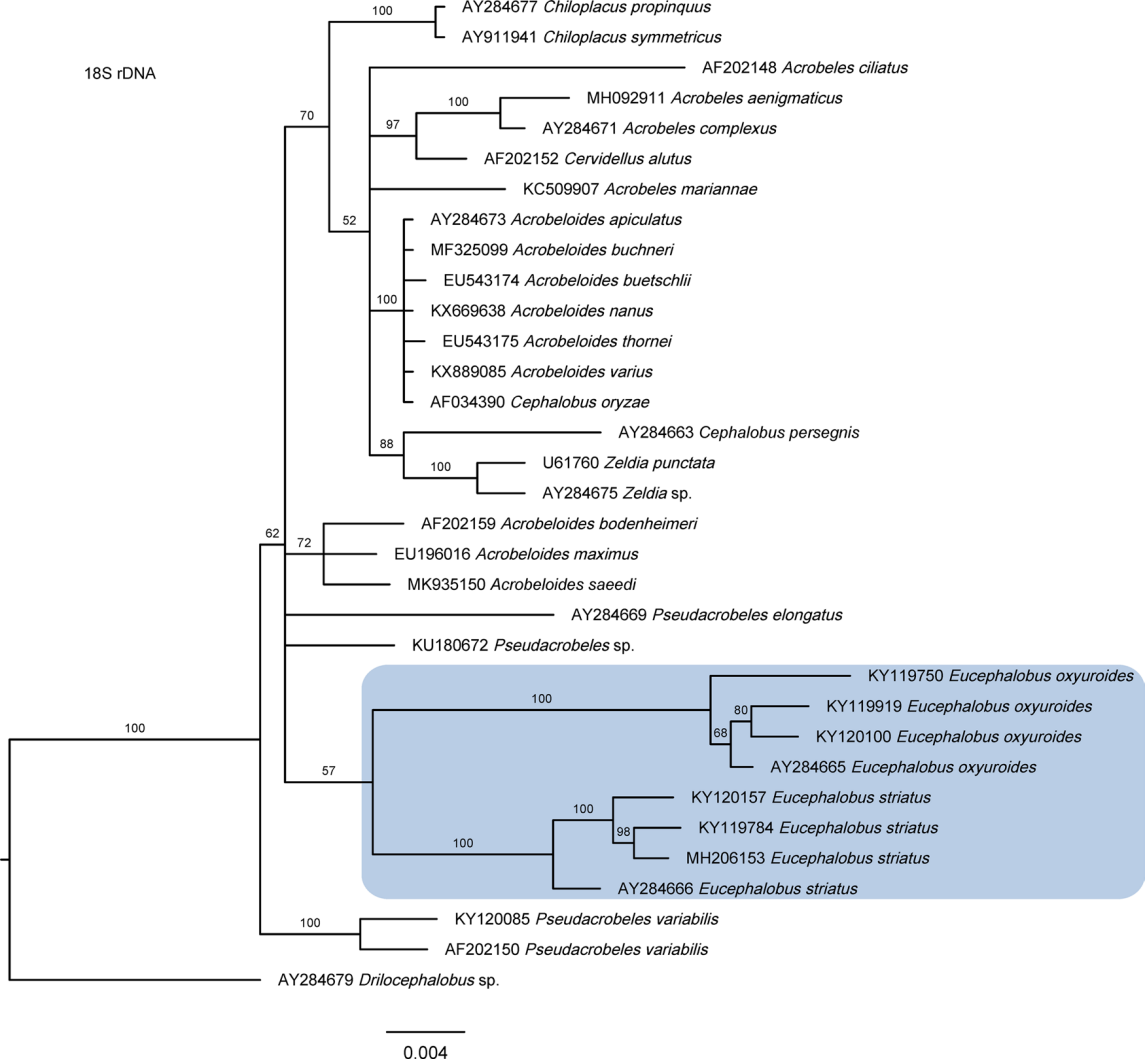
Bayesian inference tree showing the phylogenetic position of *Eucephalobus* and related taxa based on sequences of the 18S rDNA region. Bayesian posterior probabilities (%) are given for each clade. Scale bar shows the number of substitutions per site.

**Figure 7:**
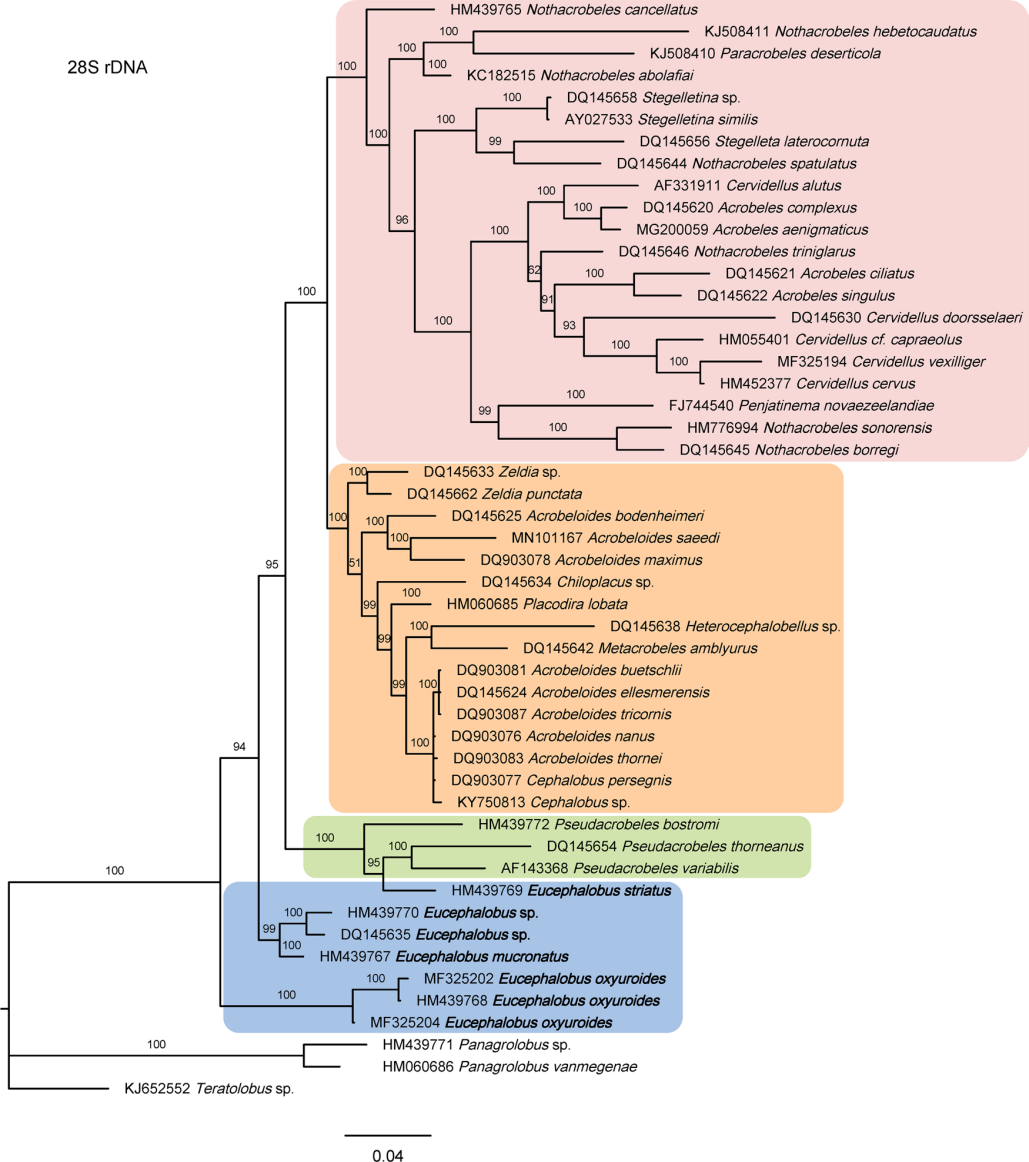
Bayesian Inference tree showing the phylogenetic position of *Eucephalobus* and related taxa based on sequences of the 28S rDNA region. Bayesian posterior probabilities (%) are given for each clade. Scale bar shows the number of substitutions per site.

As derived from the molecular analyses based on the 18S and 28S rDNA fragments, the evolutionary relationships of *Eucephalobus* are presented in the trees of Figures 6 and 7, respectively. The phylogenetic tree generated by 28S sequences is robust, however, the 18S tree is poorly resolved having Bayesian probabilities with low values. The most relevant result of these analyses is that, in both cases, *Eucephalobus* sequences occupy a basal position, together with sequences of the genus *Pseudacrobeles*. Morphologically, these two genera are very similar taxa, especially when elongate-tailed species of *Eucephalobus* – for instance, *E. oxyuroides*– are compared to members of *Pseudacrobeles*, as they share simple (poorly developed) labial probolae, probably a plesiomorphic condition within Cephalobidae against the more complex (elongate and ramified) probolae that characterizes other lineages of the family and probably represents the corresponding apomorphic condition. Thus, present results propose a new approach to interpret the Cephalobidae phylogeny, in agreement with traditional postulates based on morphological data. Previous contributions, mainly based on molecular analyses, situated *Eucephalobus* species at intermediate (rather than basal) position within the subfamily Cephalobidae, closer to either the representatives of *Zeldia* (Nadler et al., 2006) or *Cephalobus* (Smythe and Nadler, 2006; Donn et al., 2012). On this subject, our results partially agree with those of van Megen et al. (2009), who also suggest a basal position of *Eucephalobus*, closer to *Cephalobus* and *Heterocephalobus* (synonym of *Pseudacrobeles*), although the phylogeny of the group was not very satisfactorily resolved by their analyses as *Eucephalobus* sequences appeared separated into two clades.
